# Nutrition standards for the charitable food system: challenges and opportunities

**DOI:** 10.1186/s12889-022-12906-6

**Published:** 2022-03-14

**Authors:** Ronli Levi, Marlene Schwartz, Elizabeth Campbell, Katie Martin, Hilary Seligman

**Affiliations:** 1 Center for Vulnerable Populations, Department of General Internal Medicine, University of California, 1001 Potrero Avenue, San Francisco, CA 946110 USA; 2 Rudd Center for Food Policy and Obesity, Department of Human Development and Family Sciences, University of Connecticut, Hartford, CT USA; 3 Institute for Hunger Research and Solutions, Connecticut Foodshare, Wallingford, CT USA; 4 Academy of Nutrition and Dietetics, Washington, D.C. USA

**Keywords:** Nutrition, health promotion, charitable food assistance, Food banks, Food security

## Abstract

Food insecurity is associated with increased risk of chronic disease and poor dietary intake. The United States charitable food system, a complex network of food banks, pantries and congregate meal sites, provides food for millions of low-income households each year. Food banks and pantries play a critical role in supporting food security and are an important contributor to dietary intake for its clients. In recent years, there has been an increased focus on sourcing and supplying more nutritious foods within the charitable food system. Despite this, there is a lack of alignment in how the charitable food system defines and tracks the nutritional quality of food.

In 2019, Healthy Eating Research convened a panel of nutrition, charitable food system and food policy experts to create a set of evidence-based nutrition standards. Standards were developed based on a review of the literature and existing nutrition ranking systems, while also considering the operational needs and capacity of the charitable food system. The panel provided recommendations for eleven distinct food categories: fruits and vegetables, grains, protein, dairy, non-dairy alternatives, beverages, mixed dishes, processed and packaged snacks, desserts, condiments and cooking staples, and other miscellaneous items. Products are ranked into three tiers, *choose often* (green), *choose sometimes* (yellow) or *choose rarely* (red), based on designated saturated fat, sodium, and added sugar thresholds. This paper outlines the expert panel’s approach and summarizes the barriers and opportunities for implementing these standards across the charitable food system.

**Supplementary Information:**

The online version contains supplementary material available at 10.1186/s12889-022-12906-6.

## Background

Food insecurity is defined by the United States Department of Agriculture (USDA) as limited or uncertain access to adequate food [1]. In 2020, 10.5% of United States (US) households were food insecure [2]. In the US, people experiencing food insecurity often must simultaneously cope with higher levels of stress, inadequate access to healthy food, and fewer resources for taking care of their health. Thus, they are also at higher risk for poor mental and physical health, including increased rates of diet related chronic diseases such as obesity, diabetes, hypertension, and heart disease [3–5].

### Nutrition in the United States charitable food system

The US charitable food system is a complex network that includes food banks (organizations responsible for sourcing, warehousing, and distributing food to community agencies); food pantries (community agencies where individuals can pick up groceries at no cost); and congregate meal sites (community agencies where individuals are served free meals for on-site consumption, such as free dining rooms and “soup kitchens”). This network provides food for millions of low-income households each year, playing an important role in supporting household food security. Because many households experience food insecurity chronically, the charitable food system has become a regular source of food that contributes substantially to the overall dietary intake of individuals living in these households [6].

Repeated and consistent client demand for healthier food, coupled with soaring rates of obesity and diet-related chronic diseases, has resulted in an intentional movement to improve nutrition across the charitable food system [7–11]. Efforts to create a healthier inventory provide a unique opportunity for the charitable food system and public health communities to work toward a common goal of improving access to nutritious food. However, in order to evaluate progress toward this goal, it is first necessary to define how nutritional quality will be measured.

A national survey of approximately 200 food banks by MAZON found many food banks were already working toward this goal: about half reported that they utilized a system to track the nutritional quality of the food they distributed [12]. However, there was substantial heterogeneity and inconsistency among the nutrition tracking systems in use. Indeed, over the last several decades, there has been a proliferation of different approaches to measuring the nutritional quality of foods from researchers, industry, retailers, nonprofits, and regulatory bodies. The numerous - and sometimes conflicting - nutrition standards create confusion for the general population about which foods are considered “healthy.” Unsurprisingly, this was occurring in the charitable food system as well.

To address this challenge, a panel consisting of public health, nutrition and charitable food system experts was convened and funded by Healthy Eating Research (HER), a national program of the Robert Wood Johnson Foundation (RWJF) in 2019. The panel was charged with developing standards (Table 1) based on the latest scientific evidence, while simultaneously accommodating the unique challenges of implementing nutrition standards in the charitable food system. The target audiences for the resulting nutrition standards include food banks, food pantries, and other charitable food system stakeholders, including Feeding America.^1^ These recommendations are intended to guide decision-making over food sourcing, purchasing, distribution, and marketing.

**Table 1:** Healthy eating research nutrition standards^c^

Food Category	Choose Often			Choose Sometimes			Choose Rarely		
	Saturated Fat	Sodium	Added Sugars	Saturated Fat	Sodium	Added Sugars^a^	Saturated Fat	Sodium	Added Sugars
**Fruits and Vegetables**	≤ 2 g	≤230 mg	0 g	*100% juice and plain dried fruit*			≥2.5 g^b^	≥480 mg	≥12 g
				≥2.5 g^b^	231–479 mg	1–11 g			
**Grains**	*First ingredient must be whole grain AND meet following thresholds:*			≥2.5 g^b^	231–479 mg	7–11 g	≥2.5 g^b^	≥480 mg	≥12 g
	≤ 2 g	≤ 230 mg	≤ 6 g						
**Protein**	≤ 2 g	≤ 230 mg	≤ 6 g	2.5–4.5 g	231–479 mg	7–11 g	≥5 g	≥480 mg	≥12 g
**Dairy**	≤ 3 g	≤ 230 mg	0 g	3.5–6 g	231–479 mg	1–11 g	≥6.5 g	≥480 mg	≥12 g
**Non-Dairy Alternatives**	≤ 2 g	≤ 230 mg	≤ 6 g	≥2.5 g	231–479 mg	7–11 g	≥2.5 g	≥480 mg	≥12 g
**Beverages**	0 g	0 mg	0 g	0 g	1–140 mg	1–11 g	≥1 g	≥141 mg	≥12 g
**Mixed Dishes**	≤ 3 g	≤ 480 mg	≤ 6 g	3.5–6 g	481–599 mg	7–11 g	≥6.5 g	≥600 mg	≥12 g
**Processed and Packaged Snacks**	None			*If a grain is the first ingredient, it must be a whole grain AND meet following thresholds:*			≥2.5 g	≥141 mg	≥7 g
				0-2 g	0–140 mg	0–6 g			
**Desserts**	None			None			*All desserts*		
**Condiments & Cooking Staples**	*Not ranked*								
**Miscellaneous Products**	*Not ranked*								

^a^Use the added sugars value when available on the Nutrition Facts label. If it is not available, use the total sugar value. The thresholds are the same for all categories except fruits and vegetables and dairy. For both fruits and vegetables and dairy, total sugar thresholds are ≤12 g for the Choose Often tier, 13–23 g for the Choose Sometimes tier, and ≥ 24 g for the Choose Rarely tier

^b^The threshold for saturated fat is the same for the *choose sometimes* and *choose rarely* categories. All saturated fat values ≥2.5 g should be ranked as *choose sometimes*. The overall ranking is based on the lowest tier of any nutrient. Thus, a grain with 3 g of saturated fat (*choose sometimes*), 300 mg of sodium (*choose sometimes*), and 13 g of added sugar (*choose rarely*) would fall into the *choose rarel*y tier, while a grain with 3 g of saturated fat (*choose sometimes*), 300 mg of sodium (*choose sometimes*), and 10 g of added sugar (*choose sometimes*) would fall into the *choose sometimes* tier

^c^Thresholds based on serving size of the United States Nutrition Facts label

The panel report was published by Healthy Eating Research in March 2020 [13]. This paper is intended to encourage and support use of these nutrition standards at all levels of the charitable food system. We aim to describe for practitioners and stakeholders the unique characteristics of the charitable food system; the methodologic approach that guided the panel’s decision-making; and the rationale for compromises made to balance usability with scientific rigor.

### Expert panel methodology

The expert panel was co-chaired by authors HKS and MBS and included fourteen panelists. These panelists, who were selected by HER and the panel co-chairs, were chosen due to their broad range of experiences across the nutrition and charitable food sectors. To ensure balance, the chairs allotted half the panel slots to members with a depth of experience in the charitable food system, paying particular attention to include those with operational expertise and an understanding of donor perspectives and sourcing needs. As the nation’s largest network of food banks and pantries, two slots were also allotted to Feeding American representatives. The remaining slots were designated for academic researchers who could provide evidence-based recommendations and representatives from national organizations who were engaged in developing and implementing nutrition policies in the charitable food sector. Finally, geographic diversity of the panelists was an important consideration for ensuring a broad range of perspectives. To minimize conflicts of interest, panel members were only considered if they did not have any industry ties.

The panel process was organized using a similar structure to that used by other HER expert panels (see Additional file 1) [14]. Panelists met monthly for 1–1.5 h over Zoom video conference over the course of a year (February 2019–January 2020) to review and discuss the most recent nutrition science, existing nutrition standards and policies, and practical considerations of food bank operations. Each meeting was organized around a discussion of specific decision points created by the panel chairs based upon a review of the literature for each food category (see Additional file 1). At the end of each meeting, the panel chairs synthesized the topics discussed and drafted recommendations to be reviewed and voted on in the next meeting. In between meetings, panelists were asked to rank food items and provide feedback on topics discussed during the prior call via Qualtrics surveys. Results of the surveys were presented at the next meeting and used to facilitate discussion and achieve consensus. Since the new Nutrition Facts label (NFL), which introduced added sugars, was implemented during the panel process, the co-chairs also held ad-hoc meetings with a smaller subgroup of members to provide added sugar recommendations to the larger panel.

Achieving consensus was not always easy or straight-forward. Chairs were tasked with incorporating diverse feedback and finding a compromise that could be agreed upon by the majority. Despite this, consensus was an important part of ensuring the standards would meet the unique needs of the system for which it was designed. The final step in the panel process was to review all the recommendations and revise, as needed. Once the recommendations were finalized, all panel members were given the opportunity to agree or disagree with the final product. All members signed on to the final recommendations, indicating consensus. The final recommendations were subsequently reviewed by members of Feeding America’s Nutritious Revisioning Taskforce, which was comprised of Feeding America and food bank representatives whose goal was to support the refinement and adoption of these recommendations in the Feeding America network.

An early decision was to define three tiers of nutrition quality – *choose often* (green); *choose sometimes* (yellow); and *choose rarely* (red). The panel’s approach to developing the thresholds for each tier was to: 1) define the food category and align on a common definition for what should be included in that category, 2) review current evidence for that food category, and 3) build consensus for tier thresholds. After the development of tier thresholds for each product category, the panel evaluated the standards as a whole and adjusted the thresholds to create alignment across categories where appropriate. This alignment allows for ease of implementation.

The review of current evidence was not intended to be exhaustive or a systematic review of the literature. Rather, the panel set out to review existing standards within the charitable food system, nutrition standards from major retailers in the US, other widely referenced guidelines that could be applied to a charitable food system setting, and the 2015–2020 Dietary Guidelines for Americans (DGA). The panel reviewed both the academic literature and the gray literature, relying on a snowball approach to drive their search. A detailed table of the literature reviewed can be found in the Additional file 1. The panel primarily focused on reviewing and comparing standards that included quantitative thresholds for individual products. While the DGA served as the foundation for the standards, SWAP (Supporting Wellness at Pantries), an existing food bank nutrition ranking system, also became an important framework for the emerging standards [15].

### Unique attributes of the charitable food system

These nutrition standards are intended to support both upstream and downstream systems change in food banks and pantries; however, the charitable food system is unique and complex with attributes that may thwart implementation if not carefully considered. As such, the following were considered in each of the panel’s decisions: the use of food weight as a key outcome metric; the challenges in ranking mixed pallets of food; relationships with food donors; and, most importantly, limitations in the capacity of food banks and pantries. These include financial (e.g., ability to purchase foods to supplement donations); personnel (e.g., including limited staff with nutrition training and, most importantly, heavy reliance on short-term volunteers); and structural (e.g., access to refrigerator space for perishable goods) capacity.

#### Food sourcing

To improve nutrition quality in the charitable food system, it is important to understand how food is sourced. Over 60% of the food that enters the system is donated. Donations to food banks come from local and national retailers; local, regional, and national growers, manufacturers, and distributers; and community food drives. On average, another 23% is sourced from federal programs under the direction of the USDA, such as The Emergency Food Assistance Program (TEFAP) and the Commodity Supplemental Food Program (CSFP). Finally, the remaining percentage (~ 19%) is purchased using money from individual and corporate donations or grants [10]. These national averages obscure substantial heterogeneity; operations vary considerably from food bank to food bank and are influenced by a variety of factors, from available financing to staff and volunteer capacity to physical space constraints.

As manufacturing donations have declined over the last decade, the system has increased its food purchases [10] and turned to fresh produce to fill gaps. In 2017, Feeding America established seven regional produce cooperatives. These centralized facilities allow food banks across different service areas to source fresh produce more efficiently by collective negotiation of lower prices and access to a greater quantity and variety.

Food pantries rely heavily on food banks. On average, 70% of food pantry inventory originates from their local food bank [10]. However, like food banks, these national averages obscure substantial heterogeneity among food pantries. Many pantries also supplement their inventory with food purchases or local donations that do not originate at the food bank.

A set of common nutrition standards creates an opportunity to align decision making across the multiple links in the sourcing chain. They can be used as donors decide what foods to donate; food banks and food pantries decide what foods to purchase; and individuals at food pantries decide what foods to take home. In fact, there is growing empirical evidence supporting the impact of this approach on improving access to healthy foods. One recent study suggests that when food banks identify their inventory by nutrition rank, their member food pantries order significantly healthier items [16]. In another study, six pantries that implemented a nutrition ranking system significantly improved the nutritional value of their inventory [17]. Finally, a third study found that after a food pantry implemented nutrition ranking and rearranged its shelves to clearly identify healthier items, the nutritional quality of food selected by clients improved significantly [18].

Thus, food bank and pantry sourcing decisions can be driven by nutrition standards, but also provide important context for the implementation of those standards. For example, stringent policies that limit or ban the donation of less healthy products may threaten relationships with the retailers and manufacturers who provide a significant portion of a food bank’s inventory. Fear of antagonizing donors has been well documented [19, 20] and makes it difficult for food banks to turn away less healthy items. However, a recent survey suggests that donors are seldom upset by requests for healthier products [12]. Food banks that have elected to cease distributing specific items, such as baked goods, candy and sugary drinks, advise that it is important to speak to donors “early and often” and explain the rationale behind key decisions [21]. National nutrition standards can support these difficult conversations by offering an explicit rationale for requests.

#### Organizational capacity: personnel, financial, and structural

Although there is considerable diversity in how food banks and pantries operate, one commonality is a heavy reliance on volunteers, which can complicate implementation of nutrition standards. Many food donations arrive in large pallets containing dozens or hundreds of assorted products that require substantial volunteer time to sort into product categories set by the standards. Additionally, volunteers may lack the training and expertise needed to sort products correctly, especially mixed dishes that contain multiple whole food ingredients. For nutrition standards to be feasible they must be easy to implement and not require substantial additional training.

An additional challenge faced by food banks is that healthier food options often cost more per pound than less healthy alternatives [22] (e.g., brown rice vs. white rice). This can be problematic because food distribution is primarily measured and reported in pounds [23], and food banks have limited financial resources. However, in the previously referenced MAZON survey, 86% of the food banks that implemented a nutrition ranking system reported either no change or an increase in annual pounds of inventory [12].

Another capacity challenge is that many food banks and pantries have limited refrigerator and freezer space, which reduces their ability to store and distribute perishable items such as fresh or frozen fruits and vegetables. Further, perishable foods must be distributed more quickly than those that are shelf-stable, requiring food banks and pantries to establish new workflows and systems [20, 24].

#### Client food preferences

The misperception that healthier foods are not desirable is another challenge to implementing nutrition standards in a food bank or food pantry. A substantial body of research indicates that people seeking food at pantries desire healthier options [8, 25]. The largest national study on this topic, Feeding America’s Hunger in America study [7], found that clients identified produce, animal proteins, and dairy as their preferred food items. Smaller studies in New York and Connecticut had similar results. Clients prefer to receive animal proteins (e.g., meat, poultry, and fish) and produce over items of lesser nutrient value [8, 25]; support nutrition interventions (e.g., nutrition education, cooking demonstrations, and nudge strategies) in food pantries; and shift their selections when nutrition information is available [25].

### Structure of the nutrition standards

With these considerations in mind, the panel made several key decisions about how the nutrition standards would be structured. These included ranking foods into three tiers, basing the standards around nutrients of concern, (i.e., nutrients that should be limited in the diet), using serving size as the reference amount, and classifying foods into product categories.

#### Rank foods into three tiers

Nutrition standards commonly use a binary or ternary system to classify the nutritional quality of products based on defined thresholds. A three-tiered approach was chosen over a two-tiered approach for several reasons, despite the potential for more difficult implementation and messaging. First, this approach allows for stricter thresholds for the top and bottom tiers. Second, three tiers can be collapsed into two, if desired by local implementers. Third, this approach recognizes there are numerous nutritional gray areas, and that not all products can be easily classified as “healthy” or “unhealthy.”

The panel recommended a visual traffic light (green, yellow or red) or text-based (*choose often, choose sometime*s or *choose rarely*) method of communicating the three tiers. The traffic light can be easily communicated regardless of literacy level or English proficiency. There is also evidence that using traffic lights to highlight the nutrition content of products can reduce consumption of red foods [26–28]. Although there has been little research on the use of text-based descriptors as a stand-alone method, evidence shows that using a combination traffic-light and text-based approach may be an effective educational tool for consumers [29]. In addition, many food banks and pantries already use a similar stoplight-text based approach [15, 30]. The panel recommends using both approaches as complements to one another, with the option to modify according to local preference.

#### Base standards on nutrients of concern

Numerous nutrition standards or profile systems exist for ranking foods as ‘healthy’ or ‘unhealthy’ within both the charitable and larger food systems. In developing the standards, the panel favored systems that use nutrient thresholds or cut-offs because the approach is simple to operationalize and requires minimal training. Systems that use algorithms (such as the Nutrient Rich Foods Index) [31] were not considered due to their complexity.

In existing systems, nutrient thresholds center on beneficial nutrients (such as fiber), nutrients of concern (such as sodium), or a combination. To prioritize feasible implementation, the panel chose to focus on three nutrients of concern (saturated fat, sodium, and added sugars), all of which are found on the NFL. This decision is consistent with recommendations from the DGA, which limit saturated fat, sodium, and added sugars based on strong evidence that these nutrients are commonly found in dietary patterns associated with increased health risks [32]. Furthermore, foods high in these three nutrients are often associated with poor diet quality [33]. Overall food product rankings were determined by the lowest tier of any nutrient. For example, a product that is ranked *choose often* for saturated fat, *choose sometimes* for sodium, and *choose rarely* for added sugars would receive a final ranking of *choose rarely.*

Although the panel acknowledged the importance of emphasizing beneficial nutrients and encouraging the intake of under consumed nutrients, such as fiber, vitamin D, calcium, and iron, they were ultimately not included in the standards (with some exceptions described below). Algorithms that include both beneficial nutrients and nutrients to limit are more complex to implement, generally requiring additional training and/or calculations. In addition, providing credit for beneficial nutrients may encourage excessive or inappropriate use of fortification in foods that may not be otherwise considered healthy. For example, a heavily fortified cereal that is high in added sugars may be considered healthy under these algorithms, even when a general consumer or a nutrition expert would recognize them as less healthy. This inconsistent messaging creates consumer confusion.

The panel, however, created two exceptions to this general strategy. The first exception was for grains. There is significant and growing evidence to support the consumption of whole grains, although few Americans meet current DGA recommendations. Thus, the panel determined that identification of whole grains from the ingredient list was critical to the nutrition standards. Fiber content, which would have been easily identifiable on the NFL, was considered as a proxy measure of whole grain content but was rejected for two reasons: (1) there are numerous products on the market that may be fortified with functional or isolated fiber, but may not otherwise be considered healthy, and (2) there was often a thin margin between the fiber content of whole grain and non-whole grain products that made it difficult to set an appropriate threshold. While using a product ingredients list to identify a whole grain is more complicated, the panel determined that this would ultimately result in better alignment with the DGA recommendations for a healthy dietary pattern.

The second exception was for 100% juice. Using just nutrients to limit, 100% juice would always be categorized as a *choose often* food because there are no added sugars, despite scientific consensus that excessive juice intake contributes to weight gain and obesity. Thus, in these standards 100% juice is automatically categorized in the middle tier, as described in more detail below. This recommendation is consistent with recommendations from the American Academy of Pediatrics (AAP) and a recent consensus statement [34] on healthy beverage consumption in early childhood from four national health and nutrition organizations including the Academy of Nutrition and Dietetics.

#### Use serving size as reference

Thresholds for the nutrients of concern (saturated fat, sodium, and added sugars) are based on the amount in a single serving on the NFL. Although many available nutrition standards use nutrients in a standardized amount of product (e.g., nutrients per 100 g (g), or nutrients per 100 cal), these methods require calculations that create implementation barriers. The Food and Drug Administration (FDA) regulates and recently updated the information presented on the NFL. Modernizing serving sizes was included in the recent FDA update to reflect actual consumer consumption more accurately.

The decision to base the standards on the amount of specific nutrients of concern in a single serving created an unintended consequence for “100-cal packages” and other small or “fun-sized” products. Most of these products are less healthy items, such as chips and candy, but their small serving sizes mean that the levels of saturated fat, sodium, or added sugars are comparable to more healthy products packaged for a standard serving. As a result, these products could be categorized as healthier than they would be if packaged as a standard serving. The panel addressed this problem by automatically classifying all desserts into the least healthy tier (*choose rarely*) and automatically categorizing all processed and packaged snack foods into either the middle or least healthy tiers (*choose sometimes* and *choose rarely*).

#### Classify products into eleven categories

The decision to sort products into eleven categories allowed thresholds for nutrients of concern to vary by product category. This product categorization is already routinely done by many food banks and food pantries within the charitable food system, limiting panel concerns about implementation challenges. The panel started its discussion with a review of DGA categories (fruits, vegetables, grains, proteins, and dairy), which would easily align with nutrition messaging provided in other settings. Additional categories, such as mixed dishes (e.g., soups and stews), were added to reflect foods commonly donated to and distributed within the charitable food system and that often defy categorization based on a single ingredient. The eleven final product categories were (1) fruits and vegetables, (2) grains, (3) protein, (4) dairy, (5) non-dairy alternatives, (6) beverages, (7) mixed dishes, (8) processed and packaged snacks, (9) desserts, (10) condiments and cooking staples, and (11) other miscellaneous items.

Product category decisions were multifaceted. The panel sought to create as few categories as possible to limit complexity, but still allow for sensible nutrient thresholds. In some cases, combining products simplified categorization. This was particularly important for the fruit and vegetable category, as there can be confusion over the appropriate categorization of some foods (e.g., tomatoes, avocados, and cucumbers). In this case, combining fruits and vegetables into a single category simplified the nutrient threshold establishment process, because added sugars are the primary nutrient of concern for fruits (and the sodium threshold is rarely relevant), while sodium is the primary nutrient of concern for vegetables (and the added sugars threshold is rarely relevant).

Similarly, the protein category includes both animal and plant proteins. Although the nutritional composition of plant and animal proteins are very different, grouping them into one category allows users of the system to recognize the overall healthier nutrient profile of lean animal proteins and plant-based proteins compared to red meats and processed meats. Although dairy is also a good source of protein, it is typically treated as a separate meal component (e.g., milk in school meal programs and DGA) so the panel elected to keep dairy in its own category.

For other products, dividing into multiple categories simplified the ranking process and allowed for important nutrient threshold distinctions. In particular, non-dairy alternatives presented a challenge. The panel considered ranking products such as soy, almond, cashew and oat “milks” with the dairy category, the protein category, and the beverage category before ultimately deciding to establish a separate category for them. The composition and bioavailability of nutrients in non-dairy alternatives render them nutritionally dissimilar to cow’s milk, making nutrient thresholds difficult to establish when combining dairy and non-dairy foods. The panel elected not to include non-dairy alternatives in the protein category because there is significant variation in the protein content of many of these products, with some having virtually no protein at all. Finally, the panel chose not to include these products in the beverage category because of complexity in categorizing yogurt, cheese, and other foods also created from non-dairy alternatives. Thus, the final decision to place non-dairy alternatives in their own category allows for more specificity in the nutrient thresholds, accommodates culturally and medically appropriate items in the top tier, and allows for identification of more and less healthy non-dairy alternative products.

Mixed dishes, such as soups, stews, frozen dinners, and boxed meals, are common in the charitable food system. Most other nutrition standards do not include separate categories for mixed dishes because the assumption is that such foods can be categorized into a primary food group component (such as dairy, protein, or grain). However, the task of categorizing a mixed dish into a primary food group component can be challenging, particularly for untrained volunteers. The decision to create a separate mixed dishes category allows staff or volunteers to easily categorize products that do not neatly fit into another category. In addition, this decision allows for a more tailored sodium threshold. Sodium is the key nutrient of concern in most mixed dishes, and because these items are generally consumed as an entire meal rather than a meal component, the sodium threshold could be moved upward with fewer concerns about exceeding daily sodium recommendations.

The processed and packaged snacks and the desserts categories are two other examples of creating separate categories rather than grouping products into an existing food category based on their primary ingredient (e.g., cookies grouped as a grain or potato chips grouped as a vegetable). These products are consistently higher in saturated fats, sodium, and added sugars making them less healthy choices. However, product reformulations in recent years have meant that there are some processed and packaged snack foods and desserts that could end up being ranked as *choose often* in their primary ingredient category, especially if they are packaged in small serving sizes. To prevent this, the panel created two separate categories for processed and packaged snacks and desserts. Similar to the Go, Slow, Whoa system [35], no items in these categories can be categorized as *choose often*. Notably, the processed and packaged snack category does not include minimally processed foods from other categories that can be eaten as snacks, such as yogurt (dairy), apple slices (fruit), or nuts (protein). Many food banks have programs, such as backpack programs providing weekend food for school children, that prioritize small packages of shelf-stable snack items. Creating a separate category for processed and packaged snack foods allows these programs to choose healthier packaged snacks, while also acknowledging that these products should be consumed in moderation.

The panel decided to separate desserts from the processed and packaged snacks category because, unlike snacks, the consensus was that desserts are, by definition, a treat and are typically high in added sugars and saturated fat. As a result, all products in this category are automatically ranked as *choose rarely*. Although there are a number of fat-free and sugar-free products on the market, there is still reason to take caution when consuming these products. Low- or fat-free items often contain higher added sugars to compensate for flavor. In addition, many sugar-free products contain artificial sweeteners, which have unclear impacts on obesity and other health outcomes. While the panel acknowledges that desserts, when consumed in moderation, can have a place in a healthy diet, it is important to note that research shows people using the charitable food system are not generally accessing the food pantry to obtain dessert items. In fact, products such as candy, cookies, and brownies consistently are ranked at the bottom of clients’ priority lists [8, 25]. Moreover, clients at food pantries in general have greater access to desserts (and other processed and packaged snacks) than access to healthier alternatives.

The condiments & cooking staples category includes items such as vinegar, oils, butter, sugar, ketchup, sauces, salad dressing, syrup, and other products that are typically used for cooking/baking or to enhance the flavor of meals. These items are combined into one category that is not ranked; rather, a focus is placed on consumer education and other messaging strategies regarding the frequency and appropriate serving sizes for these items. There were multiple panel discussions about the advantages and disadvantages of ranking these items, especially concerning overconsumption of certain condiments like salad dressing and mayonnaise. However, research has shown that consuming food that was prepared at home is associated with improved diet quality, and the panel reasoned that not ranking these items would promote home cooking and greater consumption of otherwise healthy food items (such as salad with dressing). The panel also recognized the importance of promoting the inclusion of condiments that are often used in preparing cuisines from a range of different cultures. For example, soy sauce and fish sauce are important ingredients in many Asian cuisines, but they would likely be classified as “use rarely” in any ranking system due to the sodium content. The inclusion of culturally relevant ingredients has also been recognized as an important element of ensuring the acceptability of nutrition interventions in food banks and pantries [36]. The intention of this approach is to encourage food banks and pantries to discover the cuisines that are important to their client population and provide culturally inclusive and relevant nutrition education to support home meal preparation.

The panel also created a miscellaneous category to categorize items such as nutrition supplements and baby food. These items are meant to fulfill the nutrition needs of specific subpopulations and are therefore not ranked.

### Developing tier thresholds

Although these standards use individual nutrients as benchmarks for assessing diet quality, this does not replace the need for a holistic approach that promotes healthy dietary patterns – a key underpinning of the DGA. Aligning these standards with the DGA was an important strategy to ensure consistency between nutrition messaging received in the charitable food setting with messaging received in other settings, such as schools and federal nutrition programs (e.g., the Special Supplemental Nutrition Program for Women, Infants, and Children (WIC) and the Supplemental Nutrition Assistance Program – Education (SNAP-Ed)). Expert ranking was utilized to ensure that the items included in each tier made intuitive sense and were consistent with what most informed individuals would consider *choose often, sometimes,* and *rarely* foods. It was also important that the *choose often* foods in each category were consistent with a healthy dietary pattern outlined by the DGA. The following sections describe the basis for each nutrient threshold decision and highlight where the values were adjusted for particular categories.

#### Added sugars

Existing consumption recommendations for added sugars vary. A review of existing standards revealed that the majority of U.S. guidelines only exist for total sugars, rather than added sugars. This is largely because added sugars were only recently included on the NFL (revised 2020). The World Health Organization, and recent iterations of the DGA, recommend limiting calories from added sugars to less than 10% of total calories per day [32, 37]. For an average adult who consumes 2000 cal per day, this equates to a maximum of approximately 200 cal (50 g) of added sugars per day. The American Heart Association (AHA) has issued more conservative guidance, recommending that women limit their intake of added sugars to 100 cal (25 g) and men to 144 cal (36 g) per day [38]. In the United Kingdom (UK), the recommendation by the Scientific Advisory Committee on Nutrition (SACN) is for no more than 5% of total calorie intake to come from added sugars [39]. The goal of the current panel was to set thresholds that would limit consumption of added sugars, while still allowing for otherwise healthy food items that contain a small amount of added sugars, such as WIC-eligible breakfast cereals, whole wheat bread, and many brands of peanut butter.

In 1996, WIC introduced the 6 g per dry ounce (oz.) total sugars cap for its cereals on the basis of the role that sugar plays in the development of dental caries [40]. This standard has been upheld to this day. Although WIC guidance is based on total sugars, the fact that cereals and grain products generally do not contain naturally occurring sugars (e.g., fructose or lactose) suggests that the 6-g threshold is roughly equivalent to added sugars. This 6 g threshold served as the starting point for the discussions about added sugars for each product category. There was consensus that 6 g of added sugars were an appropriate *choose often* threshold for the grains, non-dairy alternatives, protein, and mixed dish categories. In addition to aligning with federal WIC nutrition standards, this threshold is < 20% of the recommended 50-g per day limit according to the DGAs, ensuring that all *choose often* foods are also not considered “high” in added sugars according to nutrient labeling claims.

One notable distinction is that WIC standards for total sugars in cereal products is ≤6 g per dry oz., rather than ≤6 g per serving. Although the dry oz. measurement often aligns with the serving size, there are some cereals with serving sizes larger than one dry oz. After review of items across multiple product categories, the panel came to the consensus that 6 g of added sugars per serving was still a reasonable threshold for these categories.

The panel discussed whether the added sugars threshold should be lower than 6 g for grains, proteins, and non-dairy alternatives but the consensus was to maintain a common 6 g threshold. For grains, many regular bread products contain small amounts of added sugar so it would have made it difficult to find *choose often* versions of this important staple food if the threshold were lowered. In the protein category, many nut butter products contain small amounts of added sugars. The allowance of some added sugars in this category allows the flexibility to include some nut butters (provided they meet the sodium and saturated fat limits) in the *choose often* tier. This was felt to be especially important to the panel given that nut butters, especially peanut butter, are a common staple food product distributed in the charitable food system and that natural nut butters (made up of nuts and oil only, without any added sugars) are not as readily available and are often more expensive.

There was considerable discussion around whether to allow for any added sugars in the non-dairy alternatives category. Some argued that no added sugars (i.e., zero grams) should be allowed in this category because there are numerous unsweetened non-dairy alternatives with no added sugars available on the market. However, allowing for 6 g of added sugars still excludes the vast majority of flavored non-dairy alternative products while still allowing the inclusion of some “original” non-flavored products that contain small amounts of added sugars for palatability.

In contrast, the panel decided that there should be no added sugars in the *choose often* tier for beverages, dairy, and fruits and vegetables. For the beverages category, the panel elected to emphasize water in the *choose often* tier, in alignment with DGA recommendations. Therefore, all beverages containing any added sugars were excluded from the healthiest tier. This decision recognizes the strong body of evidence that consumption of sugar sweetened beverages is linked with obesity and diet-related chronic disease [41, 42]. All diet beverages are also excluded from the highest tier on the basis of their sodium content. For the dairy category, the panel excluded all flavored milks and yogurts from the *choose often* tier, which is consistent with expert guidance from another HER panel report on healthy beverage consumption [34].

For the fruits and vegetables category, the panel elected to allow for only fresh produce and fruit canned in 100% juice or water in the *choose often* tier. As a result, no added sugars were allowed for the *choose often* tier and thresholds were set to allow for fruit packed in light syrup and heavy syrup into the *choose sometimes* and *choose rarely* tiers, respectively. There was considerable discussion around this decision as many panel members preferred to include fruit packed in light syrup in the *choose often* tier, as a way to promote fruit intake and due to often limited availability of fresh produce in some food bank settings. The panel accommodated the desire to promote fruit consumption, even in instances when fresh fruit is unavailable, by allowing canned fruit packed in 100% juice or water in the *choose often* tier.

#### Sodium

Although the DGAs recommend limiting sodium consumption to 2300 mg (mg) per day, current consumption by the average US adult (at approximately 3400 mg per day) significantly exceeds these recommendations [32]. While some public health experts have suggested that certain high-risk subpopulations (e.g., individuals with cardiovascular disease) would benefit from an even more restrictive daily sodium intake of < 1500 mg/day, the National Academy of Medicine (NAM, formerly the Institute of Medicine) has concluded a lack of evidence to support this lower threshold. Thus, the 2300 mg per day upper limit remains the general guideline [43].

For individual food products, the Daily Value (DV) for sodium guided the expert panel’s decision to set the *choose often* threshold at 230 mg sodium (10% of daily recommended intake) for all categories except beverages, processed and packaged snacks, and mixed dishes. The panel considered setting the threshold at 140 mg to align with federal regulations for low-sodium labeling, however this was felt to be overly restrictive, particularly in the fruits and vegetables category, and potentially discouraging of canned vegetable consumption if no other options are available. The 230 mg sodium threshold means that no product in the *choose often* tier is high in sodium according to nutrition labeling claims. Further, this threshold (10% of the daily value) is consistent with the sodium limit for numerous American Heart Association heart check certification products (whose guidelines range from 140 to 480 mg depending on the product) [44]. The *choose rarely* tier threshold was set at 480 mg per serving for the majority of product categories.

The exceptions to these thresholds occur in the mixed dishes, beverages, and processed and packaged snacks categories. The panel determined that no beverages in the *choose often* tier should contain any sodium, as described in detail above. Conversely, the guidance for mixed dishes was liberalized to 480 mg, because the items in this category are more likely to be consumed as an entire meal. This 480 mg threshold for the *choose often* tier is consistent with sodium guidance for the “healthy” labeling claim.

The packaged and processed snacks category, unlike the other product categories, only allows foods to be ranked as *choose sometimes* or *choose rarely.* Sodium is a nutrient of concern for many snacks, such as chips and crackers. There was clear consensus among the panel that items such as cheese puffs and flavored corn chips should be considered *choose rarely* foods*,* however there was less consensus around items such as plain popcorn and whole grain crackers. Due to a combination of small serving sizes and product reformulation, many products that the panel felt should be identified as *choose rarely* would not have been classified this way with a 240 mg sodium threshold. As a result, the panel opted to lower the threshold so that only low sodium products (those that meet the 140 mg sodium per serving threshold) would meet the criteria for *choose sometimes* in the processed and packaged snacks category and the remainder would be *choose rarely*.

#### Saturated fat

The AHA recommends that saturated fat intake be limited to < 7% of total calories for the general population and 5–6% of total calories for individuals at increased risk of cardiovascular disease [45]. The DGA recommends limiting saturated fat to < 10% of caloric intake per day [32]. These recommendations translate to approximately 16–22 g (7–10%) of saturated fat per day based on a 2000 cal diet. These general guidelines informed decisions regarding saturated fat thresholds in the individual product categories.

Saturated fat was determined to be a primary nutrient of concern for the dairy and protein categories. For dairy products, the panel concluded that non-fat and low-fat cheeses and unflavored milks should be ranked *choose often.* For protein products, they concluded that extra lean animal- and all plant-based proteins without significant amounts of added sodium should be ranked in the *choose often* tier*.* With these decisions as a guide, the panel chose to use the FDA labeling standard for extra lean meat, which allows up to 2 g of saturated fat (per 100 g), for the *choose often* tier threshold. Animal and plant proteins classified as lean based on FDA standards (up to 4.5 g of saturated fat per 100 g) were ranked in the *choose sometimes* tier*.* Although this may vary, 100 g typically correlates with a serving size of meat, poultry or fish on the NFL.

To align across product categories, the panel also used the 2 g threshold as a guide for the *choose often* saturated fat threshold in other food categories. There was agreement that this threshold was appropriate for the grains, non-dairy alternative, and fruits and vegetables categories. Although saturated fat is not a nutrient of concern commonly found in these categories, the more liberal threshold in the fruits and vegetables category, for example, allows for avocados to be ranked as *choose often*.

The categories where these recommendations diverge are dairy, mixed dishes, and beverages. For dairy, there was general consensus that unflavored nonfat yogurts and nonfat milks should be in the *choose often* tier and that full fat flavored milks and flavored yogurts with significant amounts of added sugar should be in the *choose rarely* tier. There is emerging, but still inconclusive, evidence of the cardiometabolic effects of dairy fat [46]. After considering this evidence, the panel decided to liberalize the saturated fat thresholds to allow for the inclusion of low-fat (1 and 2%) dairy products in the *choose often* tier. In addition, the panel concluded that plain, full-fat milk and yogurt should be included in the *choose sometimes* tier. Subsequently, this would also allow for the inclusion of many full-fat cheeses into the *choose sometimes* tier. Given that cheese is a major source of saturated fat in the American diet, the panel recommends that education at the pantry level focus on promoting the intake of fluid milk (rather than cheese) to meet calcium needs.

Another exception to the 2 g saturated fat threshold was in the mixed dish category, which allows for up to 3 g in the *choose often* tier and mirrors the more liberal saturated fat thresholds in the dairy category. This is due to the fact that items in this category are typically entrees and not individual food items, such as snacks or side dishes.

Finally, although saturated fat is not typically a nutrient of concern in the beverages category, the panel took a strict approach and set the saturated fat thresholds at 0 for both the *choose often* or *choose sometimes* tiers.

### Limitations and future challenges

Although the panel process has proven to be a highly cost-effective, time-efficient, and successful way to create solutions to complex problems, there are also limitations. While panel members were selected to provide broad representation and achieve balance, members may not have necessarily represented all stakeholder groups. Although the panel employed a process similar to that of other HER panels, we did not use an established decision-making framework (e.g., nominal group technique, Delphi technique) to drive our consensus building process. In addition, the recommendations were developed prior to the COVID-19 pandemic, which had a substantial impact on food bank and pantry operations. Thus, as both the nutrition science and standard charitable food system operating procedures evolve over time, it will be important to update these standards.

While the expert panel spent a considerable amount of time formulating standards for each product category, this approach focuses on comparing foods within each category, and does not adequately describe how to compare the nutritional quality of products across categories. For example, while the standards distinguish how to compare a *choose often* vegetable to a *choose rarely* vegetable, there is limited information on how to compare a *choose often* fruit to a *choose often* grain. Additional clarification is important for guiding food banks in their purchasing decisions; future efforts should focus on developing guidance that allows food banks to compare the quality of foods across categories. Guidance should emphasize purchasing decisions that align with current DGA guidelines to consume fruits and vegetables, whole grains, lean proteins, and low-fat dairy products over processed and packaged snacks and desserts. Although these standards are intended to be used by food banks and pantries to guide purchasing decisions, the *choose often, choose sometimes,* and *choose rarely* language may also be used to communicate healthy food choices to pantry users. While research has shown that ranking system can improve client food selection at pantries [18], the panel made the decision to give food banks and pantries the flexibility to tailor their nutrition education for their specific pantry population. As such, the panel did not include specific recommendations for frequency of intake. Future research should focus on testing the optimal messaging of these standards for clients.

These nutrition standards offer a first step in moving towards a common definition of “healthy” in the charitable food system. In March 2020, Feeding America formally adopted these standards and will encourage, but not require, food banks to implement them over the next five years. Implementation across a diverse and complex system presents several opportunities and challenges. First, guidance must be developed to accommodate a broad spectrum of implementation approaches based on organizational need, capability, and philosophy. Feeding America recently developed a toolkit to support implementation of these standards, which will continue to be updated over time [47]. Second, current reporting systems and metrics are designed to capture the quantity, rather than the quality, of food distributed throughout the charitable food system. Appropriate metrics will need to be developed that allow food banks and pantries to evaluate and benchmark their progress toward a healthier product mix.

## Conclusions and policy implications

People who receive food from food banks and food pantries struggle with disproportionately high rates of diet-related chronic diseases. Recognizing this, Feeding America, food banks, and food pantries around the country are prioritizing health and nutrition in their operations. Having a standard system for ranking the nutritional quality of charitable food is a major contribution to the field and will make this work easier. As food banks adopt and implement these standards, a new metric should be created based on the percentage of inventory that is in the *choose often, choose sometimes, or choose rarely* tiers to add important detail beyond the standard yet simple measure of total pounds of food.

It is critical to contextualize these efforts into the broader movement to increase access to healthy food for individuals and families experiencing food insecurity and highlight policy implications for this work. Many direct service organizations rely on food banks for a large majority of their inventory, and many food banks receive donations from manufacturers, distributors, and growers as well as the federal government. As food banks look to implement these nutrition standards, efforts should also be made to better track and monitor the quality of their inventories. These data could be used to inform policies influencing the types of food available through the emergency food network. For example, possible federal, state, and local strategies could include incentivizing the donation of healthy foods through additional tax credits, influencing the types of foods available through commodity programs, and providing transportation and storage grants. Each of these could be improved via a more thorough understanding of the types of food currently moving through the charitable food system and what product mix is needed to better align with these recommendations. An important benefit of communicating these standards to food donors is that food manufacturers may choose to alter the composition of their products to better align with the *choose often* and *choose sometimes* tiers, thus making the overall food supply healthier for all consumers.

As these standards are implemented by food banks around the US, there will be opportunities to increase efficiency and ensure that the burden of ranking does not fall entirely on the food banks. For example, federal programs like TEFAP could rank their products and make that information visible online. This would allow food banks to make informed decisions about which items to select, and it would save the time and effort of each individual food bank ranking the same products upon delivery. Similarly, other large national donors could consider ranking some or all their products prior to distribution, effectively doing the task once instead of asking food banks to repeat the same process on the same foods all over the country. Although these standards were intended for a US audience, the need for the charitable food system to address non-communicable diseases and provide nutrition in addition to calories is of global concern.

Looking to the immediate future, more work is needed to provide guidance for implementing these nutrition standards into the day-to-day operations of food banks and food pantries. Nutrition professionals, including student interns, can provide valuable assistance to help staff and volunteers translate the standards into action.

## Supplementary Information

**Additional file 1.**

## Data Availability

Slide decks with a review of the evidence are available to qualified requestors. Contact Ronli Levi (ronli.levi@ucsf.edu).

## Abbreviations

AAP: American Academy of Pediatrics
AHA: American Heart Association
CSFP: Commodity Supplemental Food Program
DV: Daily Value
DGA: Dietary Guidelines for Americans
FDA: Food and Drug Administration
g: Grams
HER: Healthy Eating Research
mg: Milligrams
NAM: National Academy of Medicine
NFL: Nutrition Facts Label
oz.: Ounce
RWJF: Robert Wood Johnson Foundation
SACN: Scientific Advisory Committee on Nutrition
WIC: Special Supplemental Nutrition Program for Women, Infants, and Children
SNAP-Ed: Supplemental Nutrition Assistance Program – Education
SWAP: Supporting Wellness at Pantries
TEFAP: The Emergency Food Assistance Program
UK: United Kingdom
US: United States
USDA: United States Department of Agriculture

## Footnotes

1 Feeding America is the United States’ largest hunger relief organization and serves an estimated 40 million people a year through its network of 200 food banks and over 60,000 food pantries and meal programs.
