# Supplementary material for: Nutrition standards for the charitable food system: challenges and opportunities
Source: BMC Public Health. 2022 Mar 14;22:495. doi: 10.1186/s12889-022-12906-6 (PMC8919136; doi:10.1186/s12889-022-12906-6)
Supplement: Supplementary file 1 — Additional file 1. [file 12889_2022_12906_MOESM1_ESM.docx]

**Methodological Appendix**

**Expert Panel Protocol**

**Background:**

Healthy Eating Research (HER) is a national program of the Robert Wood Johnson Foundation (RWJF). The program supports research on environmental and policy strategies with strong potential to promote healthy eating among children to prevent childhood obesity, especially among lower-income and racial and ethnic populations at highest risk for obesity. In 2013, HER was approached by the Partnership for a Healthier America for technical assistance on defining “healthier beverages.” They were in need of such a definition for their various partnership commitments; however, there was no uniform set of nationally available recommendations to guide them at the time. In addition to widespread public confusion regarding healthy and unhealthy beverage choices, there were a myriad of recommendations – often conflicting and inconsistent – leading to more confusion. Thus, HER convened an advisory panel of prominent researchers, nutritionists, and policy experts with expertise in nutrition and obesity prevention to develop a set of age-based recommendations defining healthier beverages. Due to limited funds, the panel met in a series of conference calls, rather than in-person meetings, to accomplish this goal. Convening a virtual, national expert panel to develop consensus recommendations proved to be a highly cost-effective, time-efficient, and successful method. HER has since convened two other expert panels and has one in progress. This document describes the HER expert panel process.

**Rationale:**

Not all research questions lend themselves well to this format. Those that do typically address a research question or gap that is timely, policy relevant, or requires consensus from the field. The benefits of an expert panel approach include:

- Allows for a multidisciplinary consensus building process
- Allows for the consolidation and augmentation of existing standards/recommendations
- Can be an important means to fill a gap in federal rulemaking/recommendation development

**Timeline:**

The process of convening a national expert panel and developing final recommendations can take anywhere from 6-12 months, depending on the research question. Review, design, and publication typically add an additional 2-4 months to this overall timeline.

**Process and Methods:**

- HER identifies key research question(s) and selects and recruits the expert panel Chair(s).
- HER develops and invites a multidisciplinary list of panelists (approximately 12-15 individuals).
- Chair(s) compile, review, analyze, and synthesize existing peer-reviewed evidence, as well as other grey literature on the topic, including evidence-based recommendations, reports, and policy statements from government, academics, industry, non-profits, and others. A preliminary document to share with panel members is developed and circulated prior to the first panel call.
- HER and the Chair(s) host virtual meetings (via conference call or video conference software, such as Zoom meeting) to review the existing evidence and discuss and develop recommendations. In general, there are about 6-8 virtual meetings. In addition, written communications and deliberations among panel members may occur between conference calls. These written communications often involve feedback on specific sections of draft recommendations or requests for additional information on a particular topic.
- Panel members with specific expertise may be tapped regarding certain recommendations or questions.
- A final set of recommendations and a narrative report are pulled together by the Chair(s) with feedback from the expert panelists and HER staff. Once all have signed off, the report enters RWJF’s editorial review and publication processes.
- HER and RWJF Communications staff work with the Chair(s) and panel members to develop an extensive communications and dissemination plan, including strategies such as webinars, email announcements, and tweetchats. In addition, depending on the recommendations, calls will be held with outside entities who may be most impacted by the recommendations to ensure there is a strong understanding of the process and the final recommendations.

**Roles and Responsibilities:**

1. **Role of the Chair, or Co-Chairs:**

- The Chair(s) will gather and analyze current evidence on the topic and existing standards prior to the first meeting of the expert panel. The information is then translated into preliminary discussion questions and/or draft recommendations prior to the first conference call in order to provide the panel members with a starting point.
- The Chair(s) will be responsible for convening a group of approximately 12-15 expert panel members with expertise in related fields. This will involve:
  - Facilitating and leading a minimum number of conference calls with expert panel members, as determined by HER;
  - Leading written communications with panel members to synthesize findings into draft recommendations; and
  - When necessary, initiating individual calls with panel members to solicit additional feedback and/or to work through concerns about or clarify final recommendations.
- The Chair(s) will draft a report (or similar document) with participation and feedback from the expert panel, which includes the expert panel’s final recommendations and related rationales for these recommendations. Upon completion, this draft will be submitted to the convener for review prior to entering the Foundation’s official editorial review and design process.
- The Chair(s) will then participate in the report review/design process by responding to edits, comments, et cetera from the convener and members/consultants of the RWJF.
- After the release of the recommendations, the Chair(s) will participate in a webinar for relevant stakeholders to present the expert panel recommendations.
- The convener will take the lead on report dissemination, however, the expert panel – including the Chair(s) – will be engaged in developing a communications/dissemination plan, and members will be expected to help disseminate the final report through their various networks and professional channels.

1. **Role of Panel Members:**

The panel will be comprised of individuals with expertise in fields relevant to the expert panel’s research question. Each panelist will serve in this capacity as a volunteer (i.e., he/she will not be paid for his/her efforts) and will be expected to:

- Participate in a minimum number of conference calls as determined by HER (each approximately one hour in length); there will be no face-to-face meetings.
- Prior to the first conference call, review all materials, including preliminary/draft recommendations and/or discussion questions sent by the panel chair(s).
- Provide input and edits on the draft standards and report throughout the panel process.
- The convener will take the lead on report dissemination; however, the expert panel will be engaged in developing a communications/dissemination plan, and members will be expected to help disseminate the final report through their various networks and professional channels.

1. **Role of Convener (i.e., HER):**

- HER will identify the panel’s primary research question(s) in consultation with RWJF.
- HER, RWJF, and the panel Chair(s) will collectively develop a list of expert panel members to be invited to participate in this effort.
- Expert panel invitations will be drafted and sent by the convener and the panel Chair(s).
- A representative from HER will assist in coordinating and scheduling expert panel meetings/conference calls.
- A representative from HER will participate in all meetings/calls of the expert panel.
- HER will review and approve draft manuscripts prior to publication and dissemination.
- A representative from HER will facilitate the review/design process by acting as the liaison between RWJF, the designers, and the expert panel Chair(s).
- HER will organize/host a webinar (or do so in partnership with another organization) to present the expert panel recommendations to researchers, advocates, and the general public after release of the final report.
- HER will take the lead on report dissemination, however, the expert panel will be engaged in developing a communications/dissemination plan, and members will be expected to help disseminate the final report through their various networks and professional channels.

**Food Bank Expert Panel Multi-Step Process:**

The development of the expert panel recommendations for developing nutrition standards for the charitable food system outlined in this report followed a multi-step process:

1. Initial discussions involving HER, RWJF, the expert panel chairs, and the panel members to gain consensus on the scope of the recommendations
2. Review of the literature on existing
3. Discussions of the literature review findings during monthly panel meetings
4. Preliminary drafting of recommendations by expert panel chairs based on evidence reviewed and panel discussions
5. Qualtrics surveys between meetings to gather input on considerations and gain consensus on recommendations
6. Review and revision of the recommendations by panel members and finalization of recommendations and best practices based on panel consensus.

| **Key Discussion Topics** |
| --- |
| **Process for standard development** |
| Determining the scope of recommendations |
| What literature/standards should be reviewed? |
| **Structure of the standards** |
| How many tiers should the standards include (e.g., two tiers, three tiers)? |
| Should the standards consider positive nutrients (e.g., calcium), negative nutrients (e.g., sodium), or both? Should foods receive “credit” for fortification? |
| Which nutrients should the thresholds be based on? Should these nutrients be the same for all food categories (e.g., should each food category include a sodium, saturated fat and added sugars threshold)? What considerations should be given for processed foods? |
| Which reference value should the thresholds be based on (e.g., per serving vs. 100 grams vs. 100 calories)? |
| What is the process for ranking a food item (e.g., should we categorize foods and rank based on the Nutrition Facts label?) |
| Who is the target audience? |
| **Defining Food Categories** |
| How many categories should be included? |
| How should each category be defined? What should be included in each category? |
| **Category Decisions** |
| ***Grains*** |
| What counts as a grain? What is the distinction between grains vs. snack foods vs. desserts vs. mixed foods? |
| Should there be a separate category for cereal (e.g., do cereals need different thresholds from the rest of the grains category due to sugar content?) |
| Should products in this category receive “credit” for whole grains? If so, how do we define whole grains (whole grain rich, first ingredient as a whole grain?) Should products be required to be whole grain to be considered for the green tier? Should there be a fiber threshold for this category? |
| ***Fruits and Vegetables*** |
| Should there be separate categories for fruits and vegetables, or should these be combined into one category? |
| Should items such as kale chips, candied fruit, and vegetables with sauce be included in the fruit and vegetable category or a separate category (e.g., as snack, dessert or mixed dish)? How should we distinguish what is included in the fruit and vegetable category vs. another category? |
| Should 100% juice be included in the fruit and vegetable category or another category (e.g., beverages)? Does juice need a separate threshold? |
| Should all fruits and vegetables fall into the “green” tier? If not, should all fresh fruits and vegetables fall into the “green” tier? Should there be separate thresholds for “processed” fruits and vegetables? What is considered a “processed” food? |
| What thresholds should we set for fruit and vegetable products with multiple ingredients (e.g., broccoli with sauce)? |
| Should any added sugar be allowed in the green category for fruits and vegetables (e.g., should fruit packed in light syrup be allowed in the green tier)? |
| ***Dairy and non-dairy alternatives*** |
| What should be included in the dairy category? Should non-dairy alternatives, dairy-based condiments/cooking staples, and dairy-based desserts be included in the dairy category? Should dairy-based beverages be included in the dairy category or beverages category? |
| Should there be separate thresholds for milk vs. cheese? |
| Should flavored milks fall into the yellow or red tier? |
| Should plain, whole milk be in the green, yellow or red tier based on the current evidence? |
| Should non-dairy products (such as almond “milk” and soy “yogurt”) have their own category or be included in existing categories (e.g., dairy, beverages)? Should soymilk be ranked with dairy products and all other non-dairy alternatives ranked with beverages? Where should other non-dairy alternatives be categorized (e.g., non-dairy “yogurt”, “cheese”)? |
| Should there be a protein threshold for non-dairy products? |
| ***Protein*** |
| Should there be separate categories/thresholds for animal vs. plant-based proteins or can these products be grouped into one category? |
| ***Processed and Packaged Snacks*** |
| How do we define “snacks” and what should this category be named? Should there be a distinction for processed and ultra processed foods? How do we define processed and packaged? |
| What is considered a snack vs. grain? Should crackers, popcorn be included in the snack category or grain category? |
| Should the “whole grain” requirement also apply to the snack category? |
| How should thresholds be defined in the context of snacks with small portion sizes (e.g., 100-calorie packs, fun sized snacks) |
| Should all snacks be ranked as red or choose rarely? Alternatively, should any snacks be considered green (e.g., should there just be a yellow and red category for snacks?) |
| Should the thresholds for the snacks category be consistent with the thresholds for the grains category? |
| ***Mixed Dishes*** |
| Should there be a separate category for mixed dishes or should items be ranked based on their primary ingredient (e.g., boxed macaroni and cheese in the grains category)? |
| What should be included in the mixed dish category vs. other categories (e.g., should boxed rice mixes be included in the grains category vs. mixed dish category?) |
| Should mixed dishes be considered a lower priority for food bank purchasing compared to other “staple” categories? How should this be communicated? |
| Should the thresholds be more lenient in this category since items typically represent entrée sized meals? |
| ***Desserts*** |
| Should desserts be included in the packaged and processed snacks category or should they be categorized separately? |
| Should there be thresholds for desserts in each tier or should all desserts be red? |
| ***Condiments and Baking Products*** |
| Should baking products be ranked? |
| Should condiments be ranked? How do we distinguish condiments from baking/cooking products? |
| Should only salad dressings and/or mayonnaise be ranked? Should there be stricter thresholds for certain condiments? |
| How do we ensure that culturally specific food items are not always ranked in the red category? |
| ***Miscellaneous*** |
| Should there be a miscellaneous items category? What should be included? |
| Should these items be ranked or not ranked? |
| **Communication of thresholds** |
| What type of naming/visual convention should be used to communicate the thresholds? |
| How should between category comparisons be communicated? |
| What are the implementation considerations for each food category? Overall? |
| How should thresholds be adjusted to align across categories? |
| How should total sugar vs. added sugar thresholds be communicated? |
| **Applying the standards** |
| How should the standards be applied across different levels of implementation (e.g., sourcing, inventory, client etc.) |

| **Literature Review References** |
| --- |
| **Nutrition and the Charitable Food System** |
| 1. An R, Wang J, Liu J, Shen J, Loehmer E, McCaffrey J. A systematic review of food pantry-based interventions in the USA. Public Health Nutr. 2019 Jun;22(9):1704–16. |
| 1. Byker Shanks C. Promoting Food Pantry Environments that Encourage Nutritious Eating Behaviors. J Acad Nutr Diet. 2017 Apr;117(4):523–5. |
| 1. Campbell E, Webb K, Crawford P. The food bank of central New York: An evaluation of the “no soda, no candy” donation policy and guests’ food preferences. Berkeley, CA: Center for Weight and Health, University of California at Berkeley; 2009. |
| 1. Campbell EC, Ross M, Webb KL. Improving the Nutritional Quality of Emergency Food: A Study of Food Bank Organizational Culture, Capacity, and Practices. 2013 Jul 3;261–80. |
| 1. Campbell E, Webb K, Ross M, Crawford P, Hudson H, Hecht K. Nutrition-Focused Food Banking. NAM Perspectives [Internet]. 2015 Apr 2; Available from: https://nam.edu/perspectives-2015-nutrition-focused-food-banking/ |
| 1. Caspi CE, Canterbury M, Carlson S, Bain J, Bohen L, Grannon K, et al. A behavioural economics approach to improving healthy food selection among food pantry clients. Public Health Nutr. 2019 Aug;22(12):2303–13. |
| 1. Dave JM, Thompson DI, Svendsen-Sanchez A, Cullen KW. Perspectives on Barriers to Eating Healthy Among Food Pantry Clients. Health Equity. 2017 Jan 1;1(1):28–34. |
| 1. Feldman M, Schwartz M. A Tipping Point: Leveraging Opportunities to Improve the Nutritional Quality of Food Bank Inventory. MAZON: A Jewish response to hunger; 2018 p. 1–18. |
| 1. Handforth B, Hennink M, Schwartz MB. A qualitative study of nutrition-based initiatives at selected food banks in the feeding America network. J Acad Nutr Diet. 2013 Mar;113(3):411–5. |
| 1. Rivera C, Alford S, Isham M, Morgan B, Just D, Swigert J, et al. The Power of Nudges: Making the Healthy Choice the Easy Choice in Food Pantries [Internet]. Feeding America; 2016. Available from: https://hungerandhealth.feedingamerica.org/resource/the-power-of-nudges-making-the-healthy-choice-the-easy-choice-in-food-pantries/ |
| 1. Robaina KA, Martin KS. Food insecurity, poor diet quality, and obesity among food pantry participants in Hartford, CT. J Nutr Educ Behav. 2013 Mar;45(2):159–64. |
| 1. Ross M, Campbell EC, Webb KL. Recent Trends in the Nutritional Quality of Food Banks’ Food and Beverage Inventory: Case Studies of Six California Food Banks. J Hunger Environ Nutr. 2013 Jul 3;8(3):294–309. |
| 1. Shimada T, Ross M, Campbell EC, Webb KL. A Model to Drive Research-Based Policy Change: Improving the Nutritional Quality of Emergency Food. J Hunger Environ Nutr. 2013 Jul 3;8(3):281–93. |
| 1. Simmet A, Depa J, Tinnemann P, Stroebele-Benschop N. The Nutritional Quality of Food Provided from Food Pantries: A Systematic Review of Existing Literature. J Acad Nutr Diet. 2017 Apr;117(4):577–88. |
| 1. Verpy H, Smith C, Reicks M. Attitudes and Behaviors of Food Donors and Perceived Needs and Wants of Food Shelf Clients. J Nutr Educ Behav. 2003 Jan 1;35(1):6–15. |
| 1. Weinfield NS, Mills G, Borger C, Gearing M, Macaluso T, Montaquila J, et al. Hunger in America 2014 National Report. Feeding America; 2014 Aug p. 177. |
| 1. Wetherill MS, White KC, Seligman H. Charitable food as prevention: Food bank leadership perspectives on food banks as agents in population health. Community Dev (Columb). 2019;50(1):92–107. |
| 1. Wetherill MS, White KC, Seligman HK. Nutrition-Focused Food Banking in the United States: A Qualitative Study of Healthy Food Distribution Initiatives. J Acad Nutr Diet. 2019 Oct;119(10):1653–65. |
| **Charitable Food System Standards** |
| 1. Byker Shanks C, Weinmann E, Holder J, McCormick M, Parks CA, Vanderwood K, et al. The UnProcessed Pantry Project Framework to Address Nutrition in the Emergency Food System. Am J Public Health. 2019 Oct;109(10):1368–70. |
| 1. Capital Area Food Bank. On the Track to Wellness [Internet]. Capital Area Food Bank. 2015. Available from: https://www.capitalareafoodbank.org/blog/2015/09/08/on-the-track-to-wellness/ |
| 1. Caspi CE, Grannon KY, Wang Q, Nanney MS, King RP. Refining and implementing the Food Assortment Scoring Tool (FAST) in food pantries. Public Health Nutr. 2018 Oct;21(14):2548–57. |
| 1. Feeding America. Feeding America’s Foods to Encourage Background [Internet]. Feeding America; 2015 Jul. Available from: http://hungerandhealth.feedingamerica.org/wp-content/uploads/legacy/mp/files/tool_and_resources/files/f2e-background-detail.v1.pdf |
| 1. Foodlink. Community Health Commitment: Nutrition guidelines for the receipt and distribution of food [Internet]. Foodlink. Available from: https://foodlinkny.org/program/food-banking/#chc |
| 1. Foodshare. SWAP [Internet]. www.foodshare.org. Available from: http://site.foodshare.org/site/PageServer?pagename=2017_programs_swap |
| 1. Helmick MJ, Yaroch AL, Parks CA, Estabrooks PA, Hill JL. Utilizing the RE-AIM framework to understand adoption of nutrition policies at food pantries across the USA. Transl Behav Med. 2019 Nov 25;9(6):1112–21. |
| 1. Martin KS, Wolff M, Callahan K, Schwartz MB. Supporting Wellness at Pantries: Development of a Nutrition Stoplight System for Food Banks and Food Pantries. J Acad Nutr Diet. 2019;119(4):553–9. |
| 1. MAZON. Choose Healthy Options Program (CHOP): Implementation Guide [Internet]. MAZON and Greater Pittsburgh Community Food Bank; Available from: https://mazon.org/assets/Uploads/HOHM-CHOPGuide.pdf |
| 1. Nanney MS, Grannon KY, Cureton C, Hoolihan C, Janowiec M, Wang Q, et al. Application of the Healthy Eating Index-2010 to the hunger relief system. Public Health Nutr. 2016 Nov;19(16):2906–14. |
| 1. PHA’s Food Assistance Partnerships: Nourishing Communities Most In-Need [Internet]. Partnership For A Healthier America. Available from: https://www.ahealthieramerica.org/articles/pha-s-food-assistance-partnerships-nourishing-communities-most-in-need-608 |
| 1. PHA’s Healthier Food and Beverage Product Calculator [Internet]. Partnership For A Healthier America. Available from: https://www.ahealthieramerica.org/articles/pha-s-healthier-food-and-beverage-product-calculator-381 |
| 1. Seidel M, Laquatra I, Woods M, Sharrard J. Applying a nutrient-rich foods index algorithm to address nutrient content of food bank food. J Acad Nutr Diet. 2015 May;115(5):695–700. |
| 1. Seidel, M., Laquatra, I., Woods, M., & Sharrard, J. (2018). Nutritional Quality Rating Systems for Food Banks and Food Pantries. Journal of the Academy of Nutrition and Dietetics, 118(11), 2043–2044. https://doi.org/10.1016/j.jand.2018.06.302 |
| **US Federal Nutrition Standards** |
| 1. U.S. Department of Health and Human Services and U.S. Department of Agriculture. 2015-2020 Dietary Guidelines for Americans. 8th Edition. [Internet]. 2015 Dec. Available from: https://health.gov/our-work/food-nutrition/2015-2020-dietary-guidelines/guidelines/ |
| 1. United States Department of Agriculture. MyPlate [Internet]. Available from: https://www.myplate.gov/ |
| 1. USDA Food and Nutrition Service. Nutrition Standards for School Meals [Internet]. Child Nutrition Programs. Available from: https://www.fns.usda.gov/cn/nutrition-standards-school-meals |
| 1. USDA Food and Nutrition Service. Smart Snacks in School [Internet]. Child Nutrition Programs. Available from: https://www.fns.usda.gov/cn/smart-snacks-school |
| 1. USDA Food and Nutrition Service. WIC Food Packages - Regulatory Requirements for WIC-Eligible Foods [Internet]. Special Supplemental Nutrition Program for Women, Infants, and Children (WIC). Available from: https://www.fns.usda.gov/wic/wic-food-packages-regulatory-requirements-wic-eligible-foods |
| **Non-US Nutrition Standards** |
| 1. Department of Health and Social Care. Guide to creating a front of pack (FoP) nutrition label for pre-packed products sold through retail outlets [Internet]. 2016 Nov. Available from: https://www.gov.uk/government/publications/front-of-pack-nutrition-labelling-guidance |
| 1. Soft Drinks Industry Levy [Internet]. GOV.UK. Available from: https://www.gov.uk/government/publications/soft-drinks-industry-levy/soft-drinks-industry-levy |
| 1. WHO guideline: Sugar Consumption Recommendation [Internet]. [cited 2020 Nov 4]. Available from: https://www.who.int/news/item/04-03-2015-who-calls-on-countries-to-reduce-sugars-intake-among-adults-and-children |
| **Other Nutrition Standards/Guidelines** |
| 1. American Heart Association. Heart-Check Certification [Internet]. www.heart.org. Available from: https://www.heart.org/en/healthy-living/company-collaboration/heart-check-certification |
| 1. Drewnowski A, Fulgoni V. Comparing the nutrient rich foods index with “Go,” “Slow,” and “Whoa,” foods. J Am Diet Assoc. 2011 Feb;111(2):280–4. |
| 1. Drewnowski A. Defining nutrient density: development and validation of the nutrient rich foods index. J Am Coll Nutr. 2009 Aug;28(4):421S-426S. |
| 1. Drewnowski A. The Nutrient Rich Foods Index helps to identify healthy, affordable foods. Am J Clin Nutr. 2010 Apr;91(4):1095S-1101S. |
| 1. Healthy Eating Research Minimum Stocking Levels and Marketing Strategies of Healthful Foods for Small Retail Food Stores. http://healthyeatingresearch.org/wp-content/uploads/2016/02/ her_minimum_stocking_final.pdf. |
| 1. Institute of Medicine (US) Committee on Examination of Front-of-Package Nutrition Rating Systems and Symbols. Front-of-Package Nutrition Rating Systems and Symbols: Phase I Report [Internet]. Wartella EA, Lichtenstein AH, Boon CS, editors. Washington (DC): National Academies Press (US); 2010. Available from: http://www.ncbi.nlm.nih.gov/books/NBK209847/ |
| 1. Institute of Medicine. Front-of-Package Nutrition Rating Systems and Symbols: Promoting Healthier Choices [Internet]. Wartella EA, Lichtenstein AH, Yaktine A, Nathan R, editors. Washington, DC: The National Academies Press; 2012. 180 p. Available from: https://www.nap.edu/catalog/13221/front-of-package-nutrition-rating-systems-and-symbols-promoting-healthier |
| 1. Lobstein T, Davies S. Defining and labelling “healthy” and “unhealthy” food. Public Health Nutr. 2009 Mar;12(3):331–40. |
| 1. National Heart, Lung, and Blood Institute. We Can! Go, Slow, and Whoa Foods [Internet]. Available from: https://www.nhlbi.nih.gov/health/educational/wecan/downloads/gswtips.pdf |
| **Industry Standards** |
| 1. Better Business Bureau. Children’s Food & Beverage Advertising Initiative [Internet]. BBB National Programs. Available from: https://bbbprograms.org/programs/all-programs/cfbai |
| 1. Guiding Stars. Guiding Stars: Science Behind the Stars [Internet]. Available from: https://guidingstars.com/ |
| 1. Kids LiveWell [Internet]. National Restaurant Association. Available from: https://restaurant.org/education-and-resources/learning-center/food-nutrition/kids-live-well/ |
| 1. Target Corporation. Target Nutritious Product Standards [Internet]. Available from: https://target.scene7.com/is/content/Target/Slingshot/2018/04/video/Nutritious_Definition_1.pdf |
| 1. The Walt Disney Company. Disney Nutrition Guidelines Criteria [Internet]. Available from: www.disneymickeycheck.com |
| 1. Walmart. Great for You [Internet]. Walmart. 2012. Available from: https://corporate.walmart.com/global-responsibility/hunger-nutrition/great-for-you |
| **Other Nutrition Considerations** |
| 1. Singhal S, Baker RD, Baker SS. A Comparison of the Nutritional Value of Cow’s Milk and Nondairy Beverages. J Pediatr Gastroenterol Nutr. 2017 May;64(5):799–805. |
| 1. Emrich TE, Qi Y, Lou WY, L’Abbe MR. Traffic-light labels could reduce population intakes of calories, total fat, saturated fat, and sodium. PLoS One [Internet]. 2017 Feb 9;12(2). Available from: https://www.ncbi.nlm.nih.gov/pmc/articles/PMC5300258/ |
| 1. Fox T, Corbett A. Better for You Foods: A Guide to Evaluating the Quality of Nutrition Standards [Internet]. Healthy Eating Research; 2018. Available from: https://healthyeatingresearch.org/research/better-for-you-foods/ |
| 1. Freedman MR, Fulgoni VL. Canned Vegetable and Fruit Consumption Is Associated with Changes in Nutrient Intake and Higher Diet Quality in Children and Adults: National Health and Nutrition Examination Survey 2001-2010. J Acad Nutr Diet. 2016 Jun;116(6):940–8. |
| 1. Johnson RK, Appel LJ, Brands M, Howard BV, Lefevre M, Lustig RH, et al. Dietary sugars intake and cardiovascular health: a scientific statement from the American Heart Association. Circulation. 2009 Sep 15;120(11):1011–20. |
| 1. Heyman MB, Abrams SA, Section on Gastroenterology, Hepatology, and Nutrition, AAP Committee on Nutrition. Fruit Juice in Infants, Children, and Adolescents: Current Recommendations. Pediatrics. 2017 Jun 1;139(6):e20170967. |
| 1. Hooper L, Martin N, Abdelhamid A, Davey Smith G. Reduction in saturated fat intake for cardiovascular disease. Cochrane Database Syst Rev. 2015 Jun 10;(6):CD011737. |
| 1. Laska MN, Pelletier JE. Minimum Stocking Levels and Marketing Strategies of Healthful Foods for Small Retail Food Stores. Durham, North Carolina: Healthy Eating Research; 2016. |
| 1. Lott M, Callahan E, Welker Duffy E, Story M, Daniels SR. Healthy Beverage Consumption in Early Childhood: Recommendations from Key National Health and Nutrition Organizations. Consensus Statement [Internet]. Durham, North Carolina: Healthy Eating Research; 2019. Available from: https://healthyeatingresearch.org/wp-content/uploads/2019/09/HER-HealthyBeverage-ConsensusStatement.pdf |
| 1. Malik VS, Popkin BM, Bray GA, Després J-P, Hu FB. Sugar Sweetened Beverages, Obesity, Type 2 Diabetes and Cardiovascular Disease risk. Circulation. 2010 Mar 23;121(11):1356–64. |
| 1. Martínez Steele E, Popkin BM, Swinburn B, Monteiro CA. The share of ultra-processed foods and the overall nutritional quality of diets in the US: evidence from a nationally representative cross-sectional study. Popul Health Metr. 2017 Feb 14;15(1):6. |
| 1. Mozaffarian D, Hao T, Rimm EB, Willett WC, Hu FB. Changes in Diet and Lifestyle and Long-Term Weight Gain in Women and Men. New England Journal of Medicine. 2011 Jun 23;364(25):2392–404. |
| 1. National Academies of Sciences, Engineering, and Medicine; Health and Medicine Division; Food and Nutrition Board; Committee to Review the Dietary Reference Intakes for Sodium and Potassium. Dietary Reference Intakes for Sodium and Potassium [Internet]. Oria M, Harrison M, Stallings VA, editors. Washington (DC): National Academies Press (US); 2019. (The National Academies Collection: Reports funded by National Institutes of Health). Available from: http://www.ncbi.nlm.nih.gov/books/NBK538102/ |
| 1. O’Neil CE, Nicklas TA, Zanovec M, Cho SS, Kleinman R. Consumption of whole grains is associated with improved diet quality and nutrient intake in children and adolescents: the National Health and Nutrition Examination Survey 1999-2004. Public Health Nutr. 2011 Feb;14(2):347–55. |
| 1. Roberto CA, Bragg MA, Schwartz MB, Seamans MJ, Musicus A, Novak N, et al. Facts up front versus traffic light food labels: a randomized controlled trial. Am J Prev Med. 2012 Aug;43(2):134–41. |
| 1. Sacks G, Veerman JL, Moodie M, Swinburn B. “Traffic-light” nutrition labelling and “junk-food” tax: a modelled comparison of cost-effectiveness for obesity prevention. Int J Obes (Lond). 2011 Jul;35(7):1001–9. |
| 1. Schulze MB, Martínez-González MA, Fung TT, Lichtenstein AH, Forouhi NG. Food based dietary patterns and chronic disease prevention. BMJ. 2018 Jun 13;361:k2396. |
| 1. Thorndike AN, Sonnenberg L, Riis J, Barraclough S, Levy DE. A 2-phase labeling and choice architecture intervention to improve healthy food and beverage choices. Am J Public Health. 2012 Mar;102(3):527–33. |
| 1. Thorndike AN, Riis J, Sonnenberg LM, Levy DE. Traffic-light labels and choice architecture: promoting healthy food choices. Am J Prev Med. 2014 Feb;46(2):143–9. |

**Expert Panel Member Bios***

********Bios at time of panel participation*

**Panel Chairs**

**Hilary Seligman, MD, MAS (co-chair)** is a Professor at the University of California San Francisco (UCSF) with appointments in the Departments of Medicine and of Epidemiology and Biostatistics. She directs the Food Policy, Health, and Hunger Research Program at UCSF’s Center for Vulnerable Populations at Zuckerberg San Francisco General Hospital and the Center for Disease Control and Prevention (CDC) Nutrition and Obesity Policy, Research and Evaluation Network (NOPREN). She also serves as Senior Medical Advisor for Feeding America. Dr. Seligman is a nationally recognized expert in food insecurity, particularly its health implications across the life course. Her policy and advocacy expertise focus on federal nutrition programs, food banking and the charitable feeding network, hunger policy, food affordability and access, and income-related drivers of food choice. Dr. Seligman founded EatSF, a healthy foods voucher program for low-income residents of San Francisco. Vouchers are redeemable for fruits and vegetables at grocery stores, corner stores, and farmer's markets in underserved neighborhoods. Outside of San Francisco, the program is known as Vouchers for Veggies. Dr. Seligman serves on the Board of Directors for California Food Policy Advocates and the San Francisco-Marin Food Bank. She also serves on the Food Security Task Force for the City and County of San Francisco. She is a Fellow of the American College of Physicians.

**Marlene Schwartz, PhD (co-chair)** is the Director for the Rudd Center for Food Policy & Obesity and a Professor of Human Development and Family Studies. Dr. Schwartz’s research and community service address how home environments, school landscapes, neighborhoods, and the media shape the eating attitudes and behaviors of children. Dr. Schwartz earned her PhD in Psychology from Yale University in 1996. Prior to joining the Rudd Center, she served as Co-Director of the Yale Center for Eating and Weight Disorders from 1996 to 2006. She has collaborated with the Connecticut State Department of Education to evaluate nutrition and physical activity policies in schools and preschools throughout the state. She co-chaired the Connecticut Obesity Task Force and has provided expert testimony on obesity-related state policies. She also serves on the Board of Directors of the Connecticut Food Bank. Dr. Schwartz has received research grants from the Robert Wood Johnson Foundation, the United States Department of Agriculture and the National Institutes of Health to study school wellness policies, the preschool nutrition environment, the effect of food marketing on children, the relationship between food insecurity and nutrition, and how federal food programs can improve the accessibility and affordability of healthy foods in low-income neighborhoods. In 2014, Dr. Schwartz received the Sarah Samuels Award from the Food and Nutrition Section of the American Public Health Association.

**Panel Conveners**

**Megan Lott, MPH, RDN** serves as Deputy Director for the Healthy Eating Research program and is based at the Duke Global Health Institute at Duke University. In this role, Megan manages day-to-day program operations, oversees coordination of scientific and administrative processes related to HER-funded research, engages in policy and advocacy collaborations with key partners, and identifies research priorities to advance program goals. Prior to coming to Duke, Megan was at The Pew Charitable Trusts in Washington, D.C. While there, she served as a Senior Associate on the Kids’ Safe and Healthful Foods Project, a collaboration between The Pew Charitable Trusts and the Robert Wood Johnson Foundation, which provides nonpartisan analysis and evidence-based recommendations on federal and state policies that affect the safety and healthfulness of school foods. In this role, Megan supported research, policy, and advocacy efforts aimed at improving the school nutrition environment, including managing the first ever Health Impact Assessment to be conducted on a federal rulemaking process. Other prior experience includes serving as the Associate Policy Director for the Community Food Security Coalition and the National Farm to School Network, where Megan managed federal and state policy initiatives, including advocating for passage of the 2010 Healthy, Hunger-Free Kids Act. Megan is a Registered Dietitian with a BS in Nutrition Sciences and Dietetics from the University of Cincinnati and a Master’s in Public Health from the University of North Carolina at Chapel Hill.

**Mary Story, PhD, RDN** is a Professor, Global Health and Family Medicine and Community Health. She also serves as the Associate Director for Academic Programs in the Institute. She brings to this position 13 years serving in leadership positions at the University of Minnesota School of Public Health, including as Senior Associate Dean for Academic and Student Affairs at the School from 2011-2013. Mary is a leading scholar in the field of child and adolescent nutrition and child obesity prevention. She has published 450 scientific articles in this field. She has received numerous national awards for her work and is a member of the National Academy of Medicine. Dr. Story has devoted her research career to the study of child and adolescent nutrition and childhood obesity. Her research has focused primarily on nutrition and diet-related issues of low-income and minority youth and their families, and environmental and behavioral community-based obesity prevention interventions for youth. Since 2005, Dr. Story has directed the Healthy Eating Research program, a national program of the Robert Wood Johnson Foundation that supports research on policy, systems and environmental strategies to promote healthy eating among children to improve nutrition and prevent childhood obesity.

**Panel Support**

**Ronli Levi, MPH, RDN (project manager)** is a Research Analyst for the Center for Vulnerable Populations at the University of California, San Francisco (UCSF), where she is involved in research and community-based interventions aimed at reducing food insecurity and improving health. Prior to joining UCSF, Ronli worked as a hospital clinical dietitian where she provided nutrition care for underserved, critically ill patients. Ronli has also worked for the Humboldt County Food Bank where she served as the education and outreach coordinator. Ronli is a registered dietitian and holds a BS in Nutritional Sciences-Dietetics from the University of Wisconsin-Madison and an MPH in Public Health Nutrition from the University of California, Berkeley.

**Kirsten Arm, MPH, RDN** is a Research Analyst for Healthy Eating Research, a national program of the Robert Wood Johnson Foundation. In this role, Kirsten manages the commissioned research portfolio, assists with review processes for HER’s funding opportunities, and provides support to HER working groups. Prior to coming to Duke, Kirsten worked as a graduate student for Healthy Eating Research at the University of Minnesota. Kirsten also worked for the Minneapolis Health Department where she provided technical assistance and education to help small food retail stores comply with the Minneapolis Staple Foods Ordinance (SFO). Kirsten is a Registered Dietitian Nutritionist and earned a BS in Community-Medical Dietetics from Viterbo University in La Crosse, WI, and an MPH degree from the University of Minnesota.

**Expert Panel**

**Gerry Brisson, MA** is President and CEO of Gleaners Community Food Bank of Southeast Michigan. A strong advocate for the community’s most vulnerable residents, he has dedicated more than 30 years of his life to improving food security. Gerry believes hunger can be solved – and the impact of addressing this problem brings greater stability, health, and empowerment to families. As president, Gerry has ushered in an era of innovation and growth at Gleaners, setting a bold vision for the food bank’s future and changing the conversation about solving hunger. Gleaners was among the first food banks in the U.S. to leverage opportunities to address the link between food insecurity and health. A business-minded and strategic leader, Gerry has pursued partnerships with leading healthcare institutions in the community and Gleaners is wrapping up its first significant pilot with Henry Ford Health System this spring. Gerry is the chair of the board for the Food Bank Council of Michigan, serving his third year in that role. A skilled and seasoned fundraiser, he has raised over $150 million to provide food for people in need during his career. He holds bachelor’s and master’s degrees in organizational management from the University of Phoenix and has earned a post-graduate certificate in fundraising management from the Center on Philanthropy at Indiana University.

**Elizabeth Campbell, MS, RDN** is a food security and nutrition consultant who focuses on advocacy, program implementation, and evaluation. As a research specialist at the University of California’s Nutrition Policy Institute (NPI) she focused on research related to improving the nutrition quality of foods distributed through the charitable food network and has helped support over 20 food banks nationally develop and implement nutrition policies. Elizabeth also worked at the Food Bank of Central New York that covers 11 counties servicing both rural and urban communities for seven years and held many positions during her tenure including Director of Internal Operations (which encompassed the warehouse and distribution management) and the Nutrition Resource Manager where she led her team’s efforts to develop and implement ‘a no soda, no candy donation policy’. At the Food Bank of Central New York, she ran the Child and Adult Care Food Program and the Summer Food Service Program. Now she is the legislative consultant for the Food Bank of Central New York and advocates on their behalf with federal representatives. In addition to her work with the emergency food network, Elizabeth has supported over 25 school districts implementing Breakfast in the Classroom as a consultant and as the Senior Program Manager at the School Nutrition Foundation. She is a registered dietitian, a member of the Academy of Nutrition and Dietetics, and the Hunger and Environment Dietetic Practice Group. Elizabeth earned her Bachelor’s degree at LeMoyne College where she majored in Human Resource Management and Industrial and Labor Relations and her master’s degree from Syracuse University where she majored in Nutrition Science.

**Gayle Carlson, MAEd** has dedicated her career to the nonprofit sector with more than 25 years as a volunteer, board member and director. In March 2013 she joined the Montana Food Bank Network as the Chief Executive Officer. Prior to her current position, she was Executive Director of the Great Falls Community Food Bank, Adjunct Professor at Great Falls College-MSU, and Director of the YWCA/Mercy Home and Big Brothers Big Sisters. Gayle holds undergraduate degrees in Legal Administration and Business Administration, a Certificate in Nonprofit Leadership and Management from Michigan State University, and a Masters in Adult Education and Distance Learning. She is a graduate of the 2014 class of Leadership Montana and 2006 Class of Leadership Great Falls. She currently serves as the Western Region Representative and Vice Chair on Feeding America’s National Advisory Council and is a member of the Missoula Sunrise Rotary. She is a proud parent of her son, 30 and daughter, 26 and doting grandma to two grandsons. She has called Montana home for 36 years but was born and raised in Michigan.

**Caitlin Caspi, ScD** is an Assistant Professor in Family Medicine and Community Health. Dr. Caspi’s research evaluates policies and interventions to address diet and weight-related health disparities. She has led a number of studies to measure and improve the nutritional quality and cultural appropriateness of food in the hunger relief system. This includes the Food Assortment Scoring Tool (FAST), a low-cost tool that food pantries can use themselves to monitor the healthfulness of the food they serve to clients. She is currently serving as the Principal Investigator on a group- randomized NIH-funded study evaluating the impact of a multi-component intervention (SuperShelf) in food pantries. SuperShelf transforms food pantries by establishing a healthy food supply and using behavioral economics to make the healthy choice the easy choice. In other research, she is evaluating the effect of a minimum wage increase on obesity and diet-related outcomes and evaluating processes to screen and intervene to address food insecurity in clinical settings. From 2016-2018 she co-led the Healthy Food Retail Methods subgroup for the RWJF Healthy Eating Research. Additionally, she serves as a founding member of the Healthy Eating and Activity Across the Lifespan (HEAL) initiative at the University of Minnesota, which has a mission to integrate research, policy, practice, and community resources to reduce weight-related health disparities

**Marla Feldman** is the Senior Program Director at MAZON, where she currently leads MAZON’s nutrition work, including its national Healthy Options, Healthy Meals™ initiative, which helps food banks implement strategies for procuring and delivering healthier food to low-income communities. During her first ten years at MAZON, Marla led the organization’s effort to strengthen California’s anti-hunger network to become leaders in advocacy and promote improved access to nutritious food. Prior to joining MAZON in 2002, Marla managed small business development grants with the African Development Foundation and the Peace Corps. Marla holds a B.A. in Communications from the University of Maryland.

**Tracy A. Fox, MPH, RDN** has over 25 years of experience working at the federal, state and local/community levels and the private sector on food and nutrition policy, legislative and regulatory processes, consensus building, collaboration, and advocacy. She is currently the president of Food, Nutrition, and Policy Consultants, LLC, where she provides policy expertise related to community health, develops evidence-based recommendations and guidelines, verifies and evaluates commitments through the Partnership for a Healthier America, and advises and counsels on policy to RWJF and other clients. Past positions have included analysts with the Department of Agriculture and the DC office of the Academy of Nutrition and Dietetics. Past and present clients include Centers for Disease Control and Prevention, Robert Wood Johnson Foundation, American Heart Association, American Cancer Society, Partnership for a Healthier America, National Head Start and public relations firms. Areas of focus include childhood and adult obesity prevention, early care and education, food insecurity, child nutrition and health, food labeling and marketing. She has presented and spoken at national, state and local venues across the country and is quoted and appears regularly in media outlets. Ms. Fox has led and participated in a number of expert advisory groups and National Academy of Medicine committees to build consensus and develop recommendations on local and state policies to prevent obesity, healthy school meals and snacks, food labeling, and sugary beverage intake in pre-school children. She has authored or co-authored numerous research reviews and expert panel recommendations and is past President of the Society for Nutrition Education and Behavior.

**Karen Hanner, MM** is the Vice President of Manufacturing Partnerships for Feeding America, the leading US hunger relief and food rescue organization. She leads a team committed to partnership development, with over 200 major manufacturers of consumer packaged goods, to ensure that unsellable food and grocery products are donated to food banks and avoid being wasted. Hanner is also a Feeding America representative on the Food Waste Reduction Alliance and led the development of the National Wasted Food Rescue Summit, hosted by Feeding America. She oversees the purchasing and disaster relief teams for the food bank network as well. Prior to joining Feeding America in 2008, Hanner held positions in marketing and supply chain management at Kraft Foods, Keebler, Nestle, and Market Day. She holds a master’s degree in management from Northwestern University and a bachelor’s of science from Georgetown University.

**Amy Headings, PhD, RDN, LDN** has been the Director of Research and Nutrition at Mid-Ohio Foodbank since 2011. She has collaborated with healthcare partners and policymakers to address food insecurity as a public health issue. She has worked with research partners to determine the impact of food access on diabetes outcomes in food-insecure populations and understand the impact of foods distributed by foodbanks on dietary intake and health outcomes in the people they serve. Amy is a registered dietitian and completed her PhD in Human Nutrition at The Ohio State University and dietetic internship at Mayo Clinic.

**Katie Martin, PhD** is the Vice President and Chief Strategy Officer at Foodshare, the regional food bank for greater Hartford, Connecticut. She has over 20 years of experience developing and evaluating creative solutions to hunger. Prior to joining Foodshare, Katie was an Assistant Professor at the University of Saint Joseph, and previously was an Assistant Research Professor at the University of Connecticut. Katie has a long track record of building collaborations with local and national anti- hunger organizations. She led the team performing the first rigorous evaluation of a food pantry program in Hartford, CT. Katie used what she learned from that project to create the “More Than Food” framework. The goal of More Than Food is to build capacity within food pantries to address underlying causes of hunger. Katie also developed a stoplight nutrition system called SWAP (Supporting Wellness at Pantries) to increase the supply and demand for healthy food in food banks and food pantries. Katie is recognized as a national leader on food security issues and has presented her research at dozens of regional and national conferences. She earned a Ph.D. in Nutritional Science & Policy from Tufts University.

**Ami McReynolds, MBA, ME** is the Chief Equity Officer at Feeding America. Ami McReynolds leads Feeding America teams supporting member relations, member grants, contract compliance, food safety, network analytics and network learning. In her role, Ami is responsible for deepening organizational understanding of network member needs and performance drivers across the Feeding America network of 200 food banks, leveraging insights grounded in data to strengthen network performance, and coordinating capacity building services, against prioritized strategies, to members. Ami joined Feeding America in January 2011 as Vice President of Talent. She became Vice President of National Programs in October 2012 and was promoted to Senior Vice President of Network Development in October, 2014. Prior to joining Feeding America, Ami spent 15 years working in for profit and higher education institutions with a focus in organizational development and leadership effectiveness. She held a variety of leadership roles in for profit companies and higher education institutions including Deloitte & Touche, Wm. Wrigley Jr. Company, a Mars Subsidiary, University of Illinois at Chicago and United Airlines. She received her undergraduate degree in Psychology and her Master’s degree in Education from Indiana University. Ami also earned her Master’s degree in Business Administration from the Mendoza College of Business at the University of Notre Dame.

**Mary Pat Raimondi MD RDN** has always had a passion for helping reduce hunger. One of her first volunteer positions was the nutrition consultant for Senator Charles Percy (R-IL) and the Senate Select committee on Hunger and Nutrition Needs. She continued volunteer work as a member of Second Harvest Heartland Board of Directors and chair of Hunger Solutions Minnesota. As the retired Vice President for Strategic Partnerships and Policy for the Academy of Nutrition and Dietetics, she represented legislative and public policy issues including food security issues in a meaningful and successful way to key stakeholders. She remains passionate about food insecurity reduction and is currently helping the Chicago Food Depository on an exciting project to help expand home-delivered meals to older adults in Chicago.

**Christina Roberto, PhD** is an Assistant Professor of Medical Ethics & Health Policy at the Perelman School of Medicine at the University of Pennsylvania. She is a psychologist and epidemiologist whose research aims to identify and understand factors that promote unhealthy eating behaviors and design interventions to improve eating habits. Christina is Director of the **P**sychology of **E**ating **A**nd **C**onsumer **H**ealth lab, or PEACH lab. In her work, she draws upon the fields of psychology, behavioral economics, epidemiology, and public health to answer research questions that can provide policymakers and institutions with science-based guidance.

**Nancy Roman, MA** is the President and CEO of the Partnership for a Healthier America after joining the organization in 2017 following an international career spanning journalism, business, and public service with the U.S. government and the United Nations. Prior to joining PHA, Ms. Roman was the President and CEO of the Capital Area Food Bank (CAFB), the Washington, D.C. region’s largest organization working to solve hunger and its companion problems: chronic undernutrition, and diet related health issues like heart disease, and obesity. Under Ms. Roman’s leadership, the food bank became a national voice for embedding health and wellness in hunger relief work. Roman currently serves on the board of trustees of Global Communities, an international NGO that works on hunger, health, micro-finance and lending to support lives and livelihoods, and of the Millennial Action Project (MAP), which organizes nonpartisan communities to find common ground on the issues facing millennials and future generations. She was recently named one of “The Most Powerful Women in Washington” by Washingtonian magazine. She holds a Master of Arts degree in International Economics and American Foreign Policy from the Johns Hopkins School of Advanced International Studies and a Bachelor of Arts degree in Journalism and French from Baylor University.

**Jennifer Seymour, PhD** is a Senior Policy Advisor and Senior Scientist in the Division of Nutrition, Physical Activity, and Obesity at the Center for Disease Control and Prevention (CDC). In this position she addresses the scientific and policy implications of nutrition and obesity interventions. Additionally, she regularly provides CDCs perspective on national and international regulations and guidance including those from FDA, USDA, HRSA, WHO, and PAHO. Dr. Seymour was the CDC lead and a member of the HHS Leadership Team for the development of the Dietary Guidelines for Americans 2020-2025 that was released in December 2020 and she has been involved in creation of the Dietary Guidelines since the 2005 edition. Dr. Seymour spent part of 2018 working at the World Health Organization on their efforts to eliminate trans fats globally. She is assisting CDC’s Division of Diabetes Translation in updating the science behind the Diabetes Prevention Program. She also has worked with USDA for many years on efforts to include public health obesity prevention efforts and policy, systems, and environmental change interventions in the SNAP-Ed program. She was the lead scientific reviewer for HBO’s 2012 Weight of the Nation documentary series and public awareness campaign and in 2010, she worked as a Policy Advisor in the Office of First Lady Michelle Obama at the White House. She assisted in planning and carrying out activities for the Let’s Move! Initiative and continued to work with Let’s Move! through 2016.
